# Optimizing County-Level Land-Use Structure Method: Case Study of W County, China

**DOI:** 10.3390/ijerph19095281

**Published:** 2022-04-26

**Authors:** Lijing Tang, Yuanyuan Yang, Dongyan Wang, Qing Wei

**Affiliations:** 1 School of Public Administration, Shandong Normal University, Jinan 250014, China; 2 Institute of Geographic Sciences and Natural Resource Research, Chinese Academy of Sciences, Beijing 100101, China; yangyy@igsnrr.ac.cn; 3 College of Earth Sciences, Jilin University, Changchun 130061, China; wang_dy@jlu.edu.cn; 4 Jintian Industry Development (Shandong) Group Co., Ltd., Jinan 250109, China; sd9986@163.com

**Keywords:** China, county level, ecological progress, land-use structure, Multiple Planning Integration, optimization

## Abstract

Planning has a direct impact on the formation of China’s land-use structure. In order to better play its role, China has proposed Multiple Planning Integration. As a part of reform for promoting ecological progress, it should have the concept of ecological progress, as well as the formation of land-use structure. Based on these, we focused on China’s land at the county level and developed a method to optimize its land-use structure catering to Multiple Planning Integration and ecological progress, using W County as a case study. This method mainly comprises three parts: calculating the demand area; calculating the carrying capacity; and optimizing the land-use structure. Models are constructed based on the ecological footprint theory. We found that setting unified targets as the link to integrating plans can effectively form the optimal land-use structure at county-level in the manner of “targets set—area determined”. There are three ways to integrate the concept of ecological progress into the optimization process. First, unified targets should be set for both ecological protection and socio-economic development, and priority should be given to the implementation of ecological protection; that is, in the process of optimization, the land area for the ecological redline of a county needs to be initially determined. Second, when optimizing the land-use structure, we should consider the carrying capacity of county-level land, in relation to demand related to the implementation of socio-economic development. Third, ecological balance should be ensured by comparing demands and the carrying capacities and maximizing the ecological service values of the land, which are important principles for determining the land-use structure. Our research provides a reference for optimizing land-use structure at the county level in China.

## 1. Introduction

Every nation needs to use its land to meet various demands. For China, planning has a direct impact on land-use. However, for a long time, administrative authorities with the planning function in the same administrative region have carried out planning based on their own responsibilities, which do not contain each other. As such, these plans have operated with the presumed use of the same land over many years; land-use conflicts have arisen objectively. To resolve these conflicts, the Chinese government has implemented Multiple Planning Integration. China’s Multiple Planning Integration reinforces the connection between different types of planning, ensuring the orderly and unified use of land, and achieves optimal land usage as one of its objectives. Since the Notification of Pilot Works of Multiple Planning Integration in Cities and Counties was issued in 2014, China’s land-use has been closely related to it.

Since the government deepened reform to promote ecological progress in 2015, ecological progress has been proposed to be integrated into every aspect in China, including, of course, the Multiple Planning Integration. This has laid the foundation that when we use land, the concept of ecological progress should be integrated. It is generally believed that the concept of ‘ecological progress’ was first coined by Iring Fetscher [1]. It is proposed to solve the facts of global resource constraints, environmental pollution and grim ecosystem degradation and its fundamental purpose is to respect and maintain the ecological environment so as to establish a harmonious symbiotic relationship between humans and nature in human progress. Ensuring ecological progress’s dominance and integrating it into socio-economic development, conforming to and protecting nature, establishing the concept of ecological protection, advocating green growth, and coordinating socio-economic development and ecological protection have been widely adopted by the scientific community as effective ways to promote ecological progress.

Objectively speaking, China’s land-use research started relatively late. It is necessary to select typical experiences of planning coordination and integration of ecological progress for land-use. From the perspective of national planning concepts, the common ideals of the European Union (EU) member states and the European Commission for the future development of the EU’s land guide their land-use decisions [2]. For example, their planning jointly specifies which places should be intensively developed and which should be protected [3,4,5]. Among the EU member states, the Netherlands has focused on the hierarchical relationships among the different planning departments to implement an orderly use of land [6,7]. The Netherlands has also emphasized the permanent protection of the “green heart” [8,9], which constrains the expansion of cities and protects green landscapes and agriculture, balancing the demands of ecological protection and the socio-economic development of land [10,11]. Similarly, the United Kingdom, Canada, and Australia have proposed “greenbelts” to limit the disorderly spread of cities and stipulate that once they are delimited, their long-term stability should be assured [12,13]. South Korea, the United Kingdom, and Mexico have also guided land-use with unified clear targets and development strategies consistent with the characteristics of the development [14,15,16,17,18,19]. Germany manages land development and construction projects through government coordination [20,21,22]. South Africa and France are focused on leading land-use through a unified land-use plan [23,24]. The USA has placed restrictions on land that needs to be protected through large-scale landscape protection measures [25], and has even used air quality management standards as a reference for land allocation [26,27]. Japan has established a unified geospatial information database to realize the sharing of planning information among the different departments [28,29]. For specific theories and technical methods, we can see that: Establishing planning targets is considered to be the premise of the selection of land-use schemes [30]; Integrating ecological planning and land planning can help bring the ecological factors and the socio-economic change process affecting the ecosystem into land-use decision-making [31,32,33]; Choosing and establishing nature reserves in a more refined way is an important way to protect ecological diversity in land-use [34,35,36,37]; Ecological conservation through land sparing is more conducive to the sharing of land-use results [38]; To increase the feasibility of land-use, constructing land-use information system is useful for providing information for planners [39,40,41], and data integrity and model methods are technical support for land-use [42,43,44,45]; Sustainable development goals can be used as a basis for solving land-use conflicts [46]; Land evaluation can make land-use produce more effective results [47,48], and the ecological assessment of land-use projects can avoid or reduce the negative impact of land-use on the ecological environment [49]; Coordination between resources and environmental carrying capacities and land-use is of great significance for land-use [50]. We can learn that the clear hierarchical relationship between planning, unified data platforms, unified land-use targets, unified land development plans, unified and appropriate technical methods, unified ecological protection scope, and balancing ecological protection and development requirements are important for reference.

For domestic research in China, the root causes of conflicts among different types of planning that hinder optimal land-use and the corresponding solutions have been examined [51,52,53]. It is widely acknowledged that “three zones and three lines” (i.e., ecological, agricultural, and urban zones) and ecological redlines, permanent basic farmland redlines, and urban development boundaries can help guide land-use in an orderly way [54,55,56]. For the practice of ecological progress, the main focus is on delineating the ecological redlines and guiding land-use with reference to resources, environmental carrying capacities, and land-use development suitability [57]. Here, we point out that China issued the Technical Guide for Delimitation of Ecological Redline [58] and carried out the demarcation of the ecological redline nationwide in 2017. The essence of the ecological redline is the bottom line of ecological security. Its delimitation is of general significance for straightening out the relationship between protection and development, improving ecosystem service functions, and building an ecological security pattern with complete structure and stable functions, which is in line with the concept of ecological progress. Ecological redline refers to all kinds of ecological elements that provide important ecological service functions and require strict protection due to their ecological sensitivity.

As we know, China’s land-use is now inseparable from the two backgrounds of Multiple Planning Integration and ecological progress, so it should be optimized based on them. Research in this area internationally has provided useful information, and many studies have been carried out at home. However, at present, there are still two issues that need to be resolved in China. First, China has always had two parallel planning systems: development planning, and spatial planning. As Multiple Planning Integration has been promoted, China has reached a consensus on integrating all plans dealing with space into land planning, but development planning will occur at the same time. Therefore, further research is needed on how to integrate the two to better guide land-use. It can be seen from international experiences, that a clear hierarchical relationship between plans is an important element in optimal land-use. In fact, the Chinese Constitution has stipulated that national economic and social development planning overrides other planning, which lays a foundation for the integration of the two. However, in practice, the dominance of national economic and social development planning is not complete, and it basically does not play a role in land-use. At the same time, the government at the national level has not yet given clear instructions on how development planning should be reflected in land-use. Therefore, we need to explore methods for the integration of development planning and land planning under this hierarchical relationship, and find methods to guide land-use in the case of their coordination. Second, we know that we should integrate ecological progress into land-use. The current literature suggests that ecological progress requires us to implement ecological protection in the process of land-use, through the ecological redline, and to align the use of land with respect to the regional carrying capacity. However, we believe that, in the era of ecological progress, ecological service values provided by land should also be implemented in land-use. Specific methodologies for these concepts in land-use need to be systematically developed.

Given the dual background of Multiple Planning Integration and ecological progress, to optimize land-use in China, resolving the two issues is urgently needed. In this paper, we take the land-use structure as the research object to explore methodologies to resolve the two issues for its optimization. As contradictions among various types of planning are concentrated at the county level, and as this level is the basic unit of ecological progress construction in China, we have mainly focused on the county level. W County was used as the case study for empirical analysis.

## 2. Theoretical Cognition

### 2.1. Integratiing Developmental and Spatial Planning for Land-Use Structure Optimization

We know that both development planning and land planning will have a direct impact on China’s land-use structure in the future. To realize optimization, the first step is to find a method to integrate them, ensuring that they can coordinate the formation of land-use structure. We start by analyzing their role in the formation of the land-use structure. Briefly, development planning focuses on putting forward socio-economic development targets. These targets can be regarded as the sum of regional socio-economic development activities in a certain period of time; that is, the development planning guides us to achieve certain targets through certain activities, which should be supported by a specific land-use structure. Land planning involves forming a specific land-use structure based on the development law of the region, according to the socio-economic development targets. Therefore, we can take the targets as the link for their integration. Setting a unified target system can impose the integrated effect of the system on the results of the land-use structure, and can help complete the optimization. Based on their hierarchical relationship, we can project the socio-economic development targets, set in development planning that are closely related to the formation of the land-use structure, onto the system. The result of the optimal land-use structure can be calculated according to the socio-economic development activities and the development law of a region. In addition, in view of the goal of county-level land planning to implement land-use targets, the target system also needs to take into account the important control targets set in land planning that China has focused on in land-use, in order to verify the land-use structure.

### 2.2. Integrating Ecological Progress in Land-Use Structure Optimization

As mentioned earlier, integrating the ecological redline, the carrying capacity and the ecological service values of land into the formation of the land-use structure with the concepts of ecological progress to help land-use structure optimization, we should find specific methodologies to implement them. For the ecological redline, since it is the bottom line of ecological security, its protection can be incorporated into the target system. At present, ecological priority has become the value orientation of land-use, as reflected in several documents such as The Opinions on Establishing a Land Planning System and Supervising Its Implementation [59]. In view of the function of the ecological redline as the bottom line, we believe that ecological priority can be implemented through it. In other words, the protection of the ecological redline should be the first factor to determine the land-use structure. For the carrying capacity, simply speaking, it is the ability to carry a region’s socio-economic development. At the same time, it is also the basis to measure whether socio-economic development is within the range of the regional carrying capacity [60,61]. Considering this, when we decide on the land-use structure, we should give full consideration to the demands of regional socio-economic development targets and the actual carrying capacity of the region. Ecological service values are the ecological services provided by land directly to humans, and they are actually the quantized values of such services [62]. Different land-use types serve different ecological service functions, so they have different quantized values. The composition of different land-use types in the regional land-use structure determines the total quantized values. What we need to do is to rationalize the composition and make the optimal land-use structure present the maximum ecological service values as much as possible.

### 2.3. Method Summarization

By summarizing the above-mentioned analysis, we mainly draw lessons from the ecological footprint theory to propose the methods in this paper. This theory was proposed by William Rees in 1992, and was perfected by his doctoral student Mathis Wackernagel [63,64]. The concept of the theory is based on converting the consumption of socio-economic activities required into areal demands for different land-use types in a region. By comparing the actual carrying capacity and the development requirements, we can judge whether socio-economic development is within the carrying capacity of the region, with biodiversity conservation areas to be deducted when calculating the carrying capacity. Through production factors and equilibrium factors, we can normalize different land-use types to carry out some targeted analysis [65,66,67].

**Figure 1:**
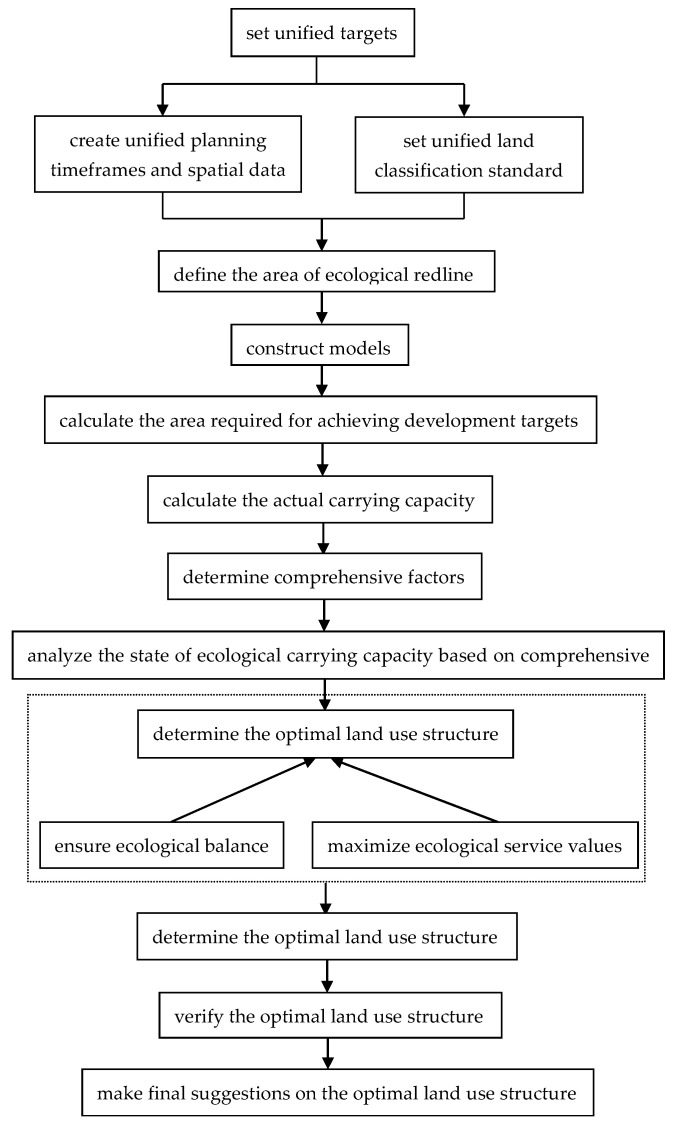
Flowchart of research methods.

Learning from the ecological footprint theory, we should first clarify the area of the ecological redline of a county. Then, we can construct models according to the corresponding relationships between socio-economic activities and different land-use types, the development law, and the accounting rules of socio-economic development, in order to calculate the areal demands of different land-use types for achieving the socio-economic development targets. After that, we need to compare the areal demands and the actual carrying capacity of various land-use types (deducting the area of the ecological redline) for the socio-economic development of a county, in order to obtain the state of the land-use structure. In addition, drawing on its normalization method, we can convert the ecological service values of different land-use types into comprehensive factors, which serve as the basis for determining the optimal land-use structure. The process of this decision is based on the principle of ensuring ecological balance and maximizing the ecological service values provided by county-level land. In order to make the optimal land-use structure more scientific, we also need to verify it with respect to the targets that have been concerned in China’s land-use, and put forward final suggestions for it. In addition, it should also be noted that the Chinese practice of Multiple Planning Integration has proved that the unification of planning timeframes, spatial data, and land classification standards is also important for the optimization of land-use structure. A flowchart of research methods is shown in Figure 1.

## 3. Research Methods

### 3.1. Basic Preparations

#### 3.1.1. Constructing a Target System

Ecological progress requires us to first implement the protection of the ecological redline, when we determine the land-use structure. After that, we can use the remaining land to implement socio-economic development. The national economic and social development planning reflects the targets for socio-economic development in a region over five years. To promote the integration of developmental and spatial planning, we should incorporate targets that are set in the national economic and social development planning, that are closely related to the adoption of land-use structure into the system. It is clear that the population and the economy are the most basic and important issues in the process of socio-economic development and are motivating forces for land-use [68], so we select targets reflecting what is needed for their development.

After a lot of thought about the targets set by the national economic and social development planning, in terms of population, the total population and the urbanization rate comprise an important basis for determining land-use structure, which can then help us obtain the demand area of different land-use types for human life. In terms of economy, the gross domestic product (GDP) has always been an important tool for measuring economic development, which can help us to obtain the demand area of different land-use types for three industrial developments. Projecting these targets onto the demand area of different land-use types, based on the population and economic development activities and the development law of a county, can help form a structural effect. Therefore, the current study incorporates total population, urbanization rate, and GDP into the target system. In addition, we consider that China has always adhered to the strictest cultivated land protection rules in the world and focused on economical and intensive land-use for construction, so, we incorporated the minimum cultivated land area and the total area of construction land set in land planning into the target system, in order to verify the land-use structure. The target system is shown in Table 1.

#### 3.1.2. Creating Uniform Planning Timeframes and Spatial Data

Due to the rapid changes and great uncertainty of national economic and social development, the planning timeframe for development planning is five years. Considering the dominant role given by the Chinese Constitution to the national economic and social development planning relative to other planning and the original intention to promote the integration of developmental and spatial planning, as well as the requirement to reflect the development targets set in the target system into land-use, we recommend that the timeframe for optimizing the county-level land-use structure should also be five years. Furthermore, to improve the convergence of planning at different levels in China, and to coordinate the management of land in the future, we recommend that, when we optimize the land-use structure, the CGCS 2000 and the 1985 National Elevation Datum should be used as common spatial data.

#### 3.1.3. Defining Land Classification Standard

Corresponding to the implementation of the target system, we first differentiated county-level land into two classifications—ecological redline and socio-economic development land. Other classification levels lie below these in the hierarchy. The classifications were based on the results of the third National Land Survey and various planning guidelines, including the Land-use Present Situation Classification (GB/T 21010-2017) [69], Guide to Land-use Classification in Spatial Planning [70], and Guidelines for the Zoning and Use Classification of Land Space Planning in Cities and Counties [71]. The land classification standard is shown in Table 2.

### 3.2. Method Flow

#### 3.2.1. Determining the Area of Ecological Redline

The Guide to Delimitation of Ecological Redline [58] has pointed out that determining the ecological redline should be synchronized with spatial identification. The associated steps are as follows: (1) evaluate the intensity of the ecosystem service function and the sensitivity of the ecological environment in the county using the relevant technical methods in the Guidelines for the Specification of the Ecological Redline; (2) specify the ecological elements in the county guidelines that must be protected, such as national parks, nature reserves, and forest parks; and (3) determine the final ecological redline by superimposing the above evaluation results, the ecological elements that must be protected, and the ecological protection options of the county.

#### 3.2.2. Determining the Demand for Land to Realize Socio-Economic Development

The ecological footprint theory converts various socio-economic activities carried out by a specific number of people in a region into different land-use types required to complete these activities, so as to judge whether each land-use type in the region can carry the realization of corresponding activities. Then, using factors to normalize different land-use types, we can know whether the regional development is within the carrying range. Inspired by this theory, we construct models to calculate the demand area of different land-use types to realize socio-economic development targets.

1. Calculating the Demand for Land to Realize GDP

GDP is composed of the output value of economic development activities from primary, secondary, and tertiary industries, which is reflected in different land-use types. Therefore, we can construct models based on the corresponding relationships between different land-use types and industries. The details are as follows: Cultivated land and garden land carry agricultural development; forest land carries the development of forestry; and cultivated land and grassland carry the development of animal husbandry. Ditches, pit-ponds, reservoirs, rivers, lake surfaces, and inland beaches, and coastal beaches carry the development of fisheries. Construction land carries the development of secondary and tertiary industries. As the output value of primary industry is closely related to direct products from the land, the demand area for primary industry development is calculated based on products. However, secondary and tertiary industries are closely related to the benefit of construction land per unit area, and the demand area of construction land is calculated based on it. The formulas are shown in Table 3.

It should be noted that the area demand for cultivated land products grown in rotation should be calculated separately, and the maximum value should be included in the cultivated land area demand. Ditches and pit-ponds are included in the water area.

2. Calculating the Demand for Land to Realize Population Development

The demand area for realizing population development was calculated using a comprehensive forecasting method, including two steps. First, we built models to calculate the demand area from the perspective of different land-use types that directly support human survival and living activities. The models were applied to cultivated land, garden land, forest land, grassland, ditches, pit-ponds, water areas, and urban and rural construction land. The second step considered the land required for key projects, such as transportation, water conservancy facilities, and other construction facilities. This is generally used according to higher-level planning decisions, relevant professional specifications, and/or the special requirements of development. Therefore, the demand area of land can be predicted based on the arrangement of key projects. The formulas are shown in Table 4.

It should be noted that the area demands for cultivated land, garden land, grassland, and water area are calculated under the assumption that all consumption in the county is completely self-sufficient. The area demand for forest land is calculated under the condition of achieving a carbon and oxygen balance in the county. The area demand for construction land is calculated from the perspective of matching people to land. Ditches and pit-ponds are included in the water area.

3. Calculating the Demand for Land to Realize Socio-Economic Development

GDP and population are decisive factors that determine the land area demand. To consider their influence comprehensively, we need to know their weights. In order to avoid the subjectivity of weighting, we used Formulas (13) and (14) to objectively determine the weights, based on the principles of the traction effect of superior factors and the constraint effect of inferior factors. Formula (15) was used to calculate the demand area:

(13) $$MaxS_{z}=w_{1}•s_{z1}+w_{2}•s_{z2}+w_{3}\sqrt[{2}]{s_{z1}•s_{z2}}$$

Constraint:

$$\left\{ \begin{array}{l} \alpha \leq {|w}_{i}-w_{j}|\leq \beta \\ \alpha ,\beta \;are\;upper-lower\;limits\;of\;weight\;difference\\ r<w_{i}<1,\left(i,j=1,2,3\;and\;i\neq j\right),r\;is\;lower\;limit\;of\;weight\\ \sum_{i=1}^{3}w_{i}=1 \end{array}\right.$$

(14) $$MinS_{z}=w_{1}•s_{z1}+w_{2}•s_{z2}+w_{3}\sqrt[{2}]{s_{z1}•s_{z2}}$$

Constraint:

(15) $$\left\{ \begin{array}{l} \alpha \leq {|w}_{i}-w_{j}|\leq \beta \\ \alpha ,\beta \;are\;upper-lower\;limits\;of\;weight\;difference\\ r<w_{i}<1,\left(i,j=1,2,3\;and\;i\neq j\right),r\;is\;lower\;limit\;of\;weight\\ \sum_{i=1}^{3}w_{i}=1 \end{array}\right.$$

where $MaxS_{z}$ represents the area demand for the z-th land-use type under the principle of the traction effect of superior factors (ha); $MinS_{z}$ represents the area demand for the z-th land-use type under the principle of the constraint effect of inferior factors (ha); $w_{i}$ and $w_{j}$ represent the weights for economic and population development targets, respectively; $s_{z1}$ and $s_{z2}$ represents the area demand for the z-th land-use type determined by GDP and population (ha), respectively.

#### 3.2.3. Determining the Carrying Capacity of Land

To calculate the carrying capacity of land to meet socio-economic development, first, we must know the current land-use situation in the county. Then, according to various land-use plans in the planning period, we can obtain the carrying capacities of different land-use types at the end of the planning period. We mentioned earlier, in order to better practice ecological progress and implement effective ecological protection, in the current study, we recommend that land for the ecological redline not be included in the calculation of the carrying capacity.

#### 3.2.4. Determining the Optimal Land-Use Structure

In this article, we draw lessons from the ecological footprint method, which uses factors to normalize different land-use types, to bring the ecological service values of land into the decision-making of optimizing land-use structure. We regard the land in each county as an open ecosystem that provides ecological service values for the county and the overall environment. So, we applied the equivalence value of ecological service values per unit area of Chinese ecosystems, determined by Xie et al., as the equivalence factor of ecological service values [72]. This reflects the relative ability of each land-use type to provide ecological service values in the county. We also took the ratio of the ecological service values per unit area for each land-use type in the county to that of the corresponding land-use type in China, as a regulation factor to reflect the relative ability of each land-use type in the county to provide ecological service values to the overall environment. The product of these two factors was used as the comprehensive factor for ecological service values to optimize the land-use structure.

Formulas (16) and (17) were used to calculate the ecological footprint and ecological carrying capacity, based on the comprehensive ecological service values:

(16) $$S_{z}=S_{z}{}^{\prime }\times x_{a}\times x_{b}$$

(17) $$A_{z}=A_{z}{}^{\prime }\times x_{a}\times x_{b}$$

where $S_{z}$ and $A_{z}$ represent the ecological footprint and ecological carrying capacity of the z-th land-use type (ha), respectively; *z* represents the z-th land-use type; $S_{z}{}^{\prime }$ and $A_{z}{}^{\prime }$ represents the area demand and actual area of the z-th land-use type (ha), respectively; $x_{a}$ and $x_{b}$ represent the equivalence factor and regulation factor for the ecological service values of the z-th land-use type, respectively.

After the calculation, the optimal land-use structure can be determined as follows: First, the ecological footprint generated by realizing socio-economic development is compared with the actual ecological carrying capacity in the county. Second, we analyze whether each land-use type is in a state of ecological balance, ecological surplus, or ecological deficit, as well as the state of the whole county. To determine the optimal land-use structure, we should first ensure the ecological balance of the county. Thus, we cannot deal with land-use types with ecological balance. For land-use types with ecological deficit, we need to put forward suggestions for the conversion of different land-use types, according to the actual situation of the county. The conversion is mainly based on the comprehensive ecological service values (reflecting the ecological service values) of different land-use types and the principle of ensuring that the land in the county presents the maximum ecological service values; that is, land-use types in a state of ecological surplus in the county and with comprehensive ecological service values from lower to higher should be adjusted successively to those with a state of ecological deficit and comprehensive ecological service values from higher to lower, until each land-use type presents ecological balance (or, even, ecological surplus). At this time, the optimal land-use structure is basically determined, and we then check it with respect to the relevant targets, and finally put forward final suggestions for the optimal land-use structure.

## 4. Results

### 4.1. Data Source and Data Processing

We considered W County in China as a case study. The 14th Five-Year Plan period covers the first five years of the country’s new journey towards building a modern socialist country with a prosperous society. Therefore, this study, we optimized the land-use structure in the 14th Five-Year Plan for W County. The basic data used are summarized in Table 5.

Spatial analysis was carried out using ArcGIS. The CGCS 2000 and the 1985 National Elevation Datum were adopted as vector data. As agricultural production is affected by interannual climate fluctuations, we took the average value of the per-unit area yield from 2016 to 2020 for each product that contributed to the primary industry output value as its per-unit area yield in the target planning year. The per-capita consumption of primary industry products in the target planning year was predicted using the GM (1,1) method, based on the relevant data in the Statistical Yearbook from 2016 to 2020. To eliminate the influence of price changes, all price and economic development target data in this paper used the constant price in 2015. LINGO was used to calculate the demand area of different land-use types to realize socio-economic development.

### 4.2. Ecological Redline Area in W County

**Figure 2:**
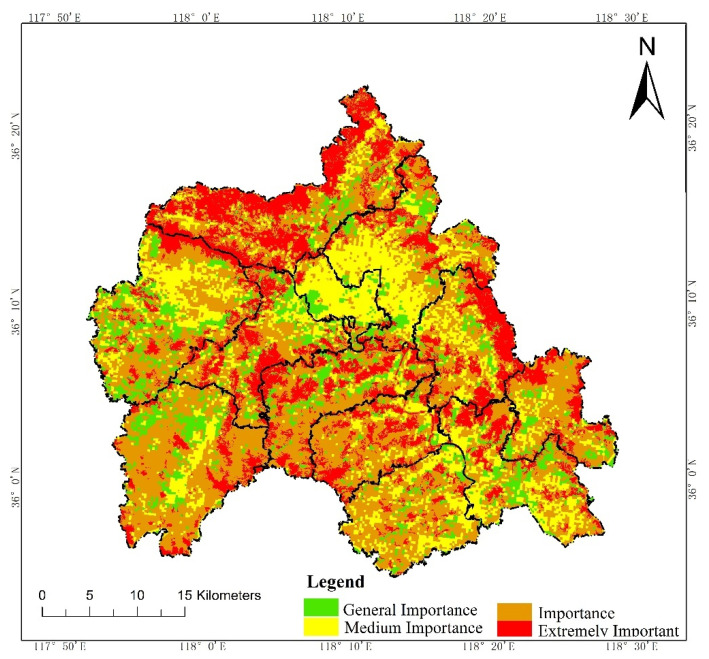
Evaluation of intensity of ecosystem service function.

**Figure 3:**
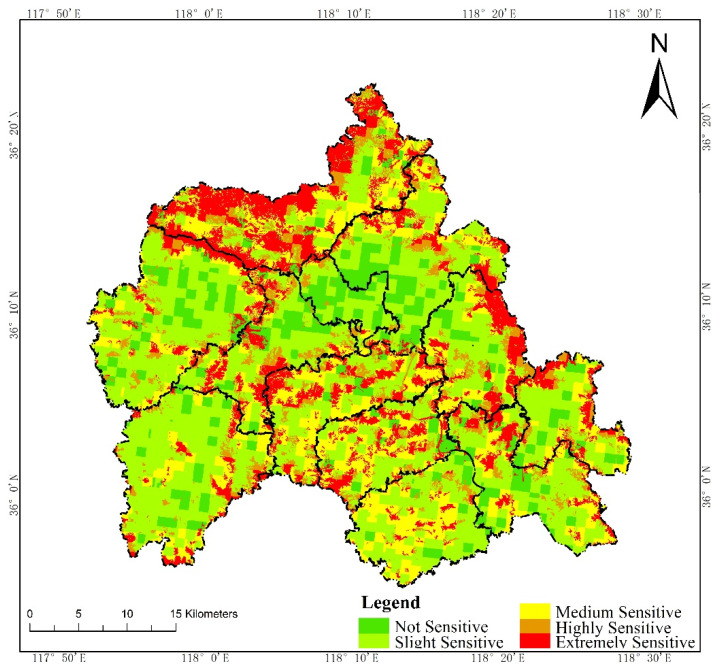
Evaluation of sensitivity level of ecological environment.

**Figure 4:**
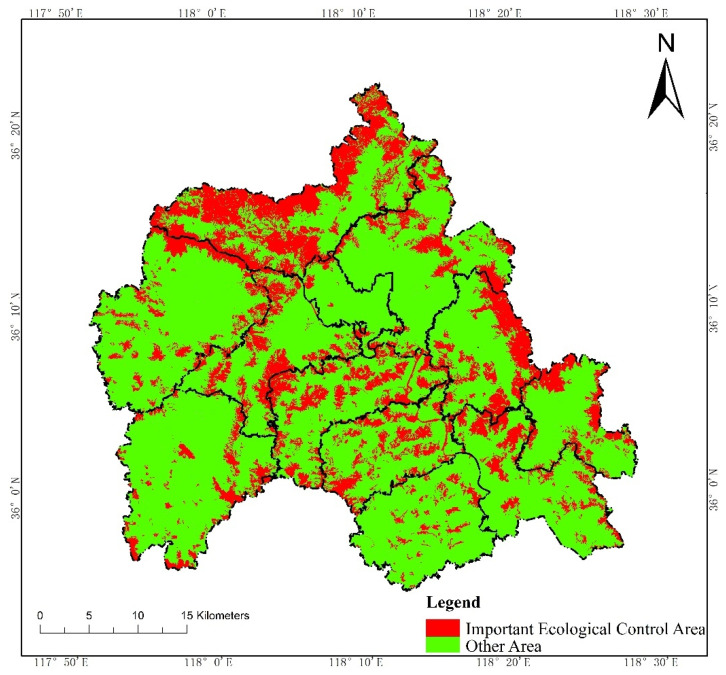
Comprehensive evaluation of ecological protection.

**Figure 5:**
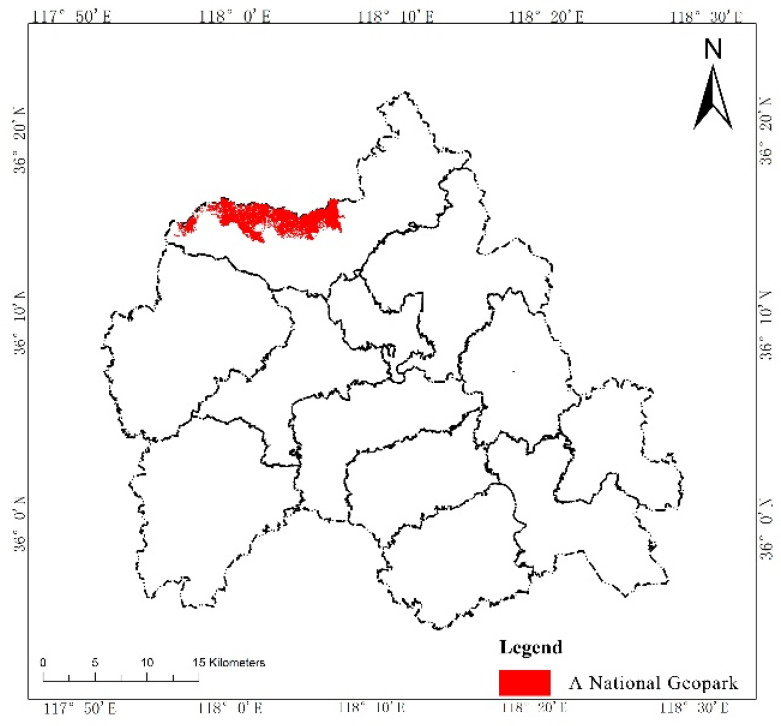
A national geopark.

**Figure 6:**
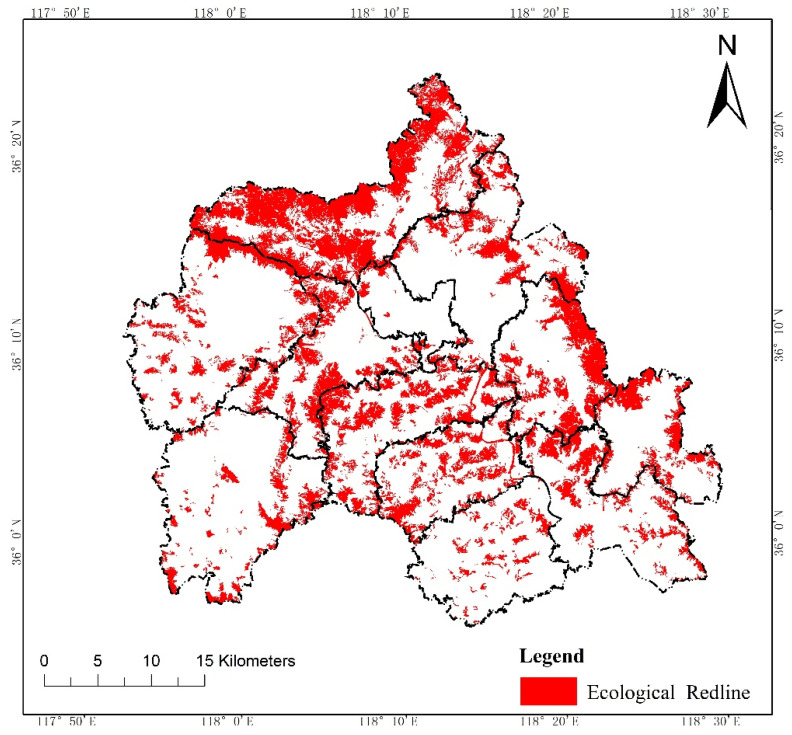
Ecological redline.

According to the Technical Guide for Delimitation of Ecological Redline, we first evaluated the intensity of the ecosystem service functions and the sensitivity level of the ecological environment in W County, based on the third National Land Survey Database (Figure 2 and Figure 3). Then, we initially delimited land with important ecosystem service functions and high ecological sensitivity in the county to the ecological redline (Figure 4). As an example of an ecological element that must be regulated, a national geopark is shown (Figure 5), with six such elements in W County. The initial ecological redline, superimposing all ecological elements that must be regulated, identified an area of 41,319.28 ha in W County (Figure 6).

### 4.3. Area Demand to Realize Socio-Economic Development in W County

#### 4.3.1. Area Demand to Realize GDP

1. Proportion of Each Product Contributing to Primary Industry Output Value

**Figure 7:**
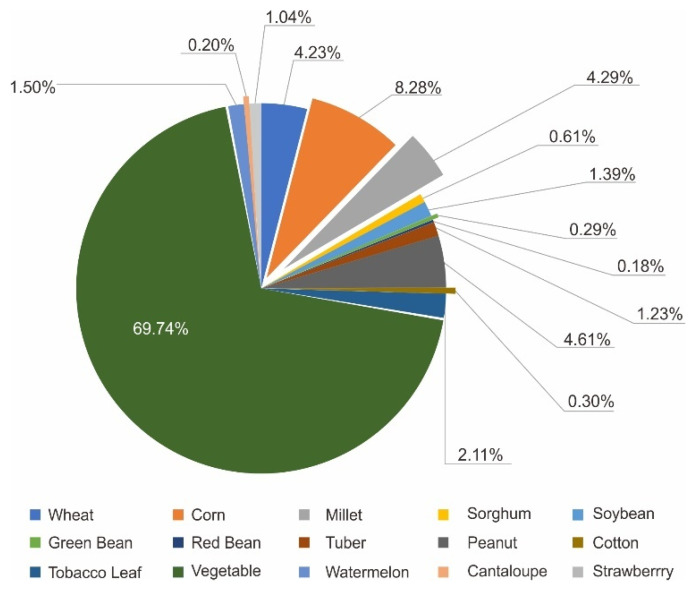
Proportions of product contributing to agriculture carried by cultivated land.

**Figure 8:**
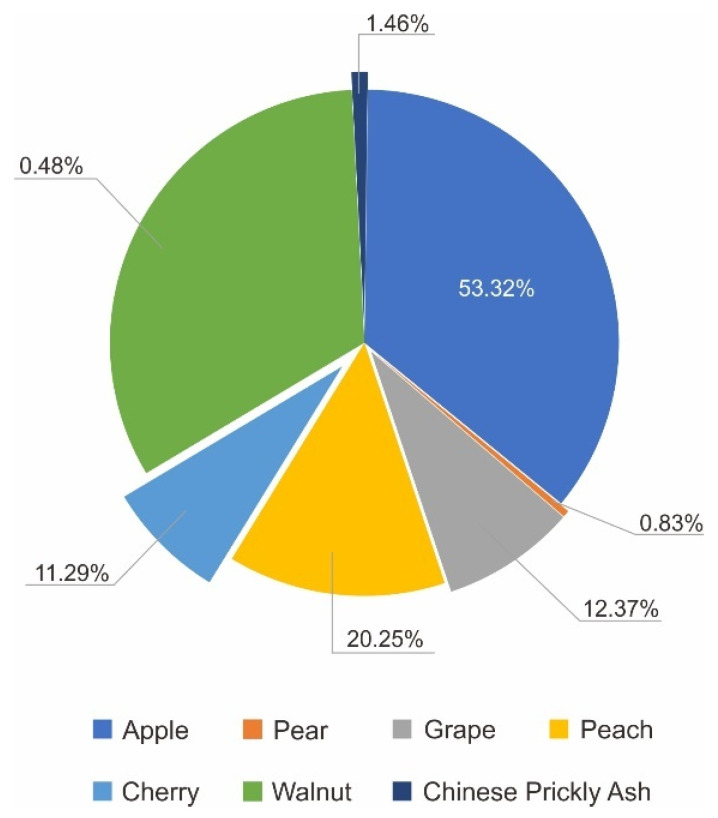
Proportions of product contributing to agriculture carried by garden land.

**Figure 9:**
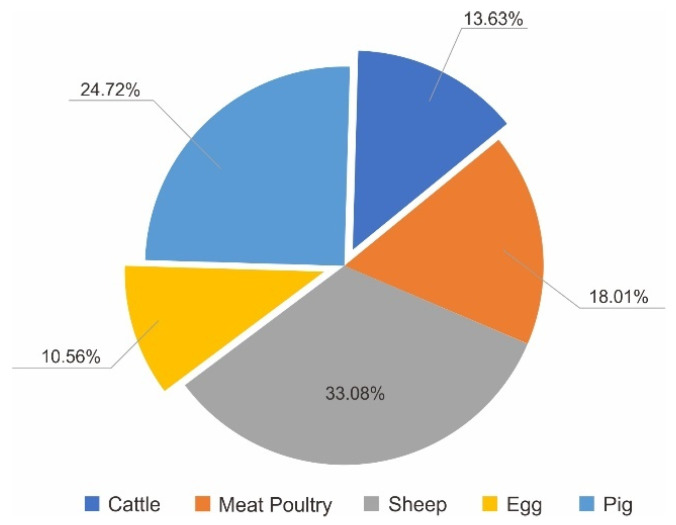
Proportions of product contributing to animal husbandry.

The proportion of each product contributing to the total output value of primary industry was calculated based on their contribution from 2016 to 2020. We used the average value over the five years. The output value for agriculture carried by cultivated land, and agriculture carried by garden land, forestry, animal husbandry, and fishery in W County accounted for 18.24%, 52.47%, 4.30%, 23.15% and 1.84%, respectively, the proportion of internal products is shown in Figure 7, Figure 8 and Figure 9, respectively. All aquatic products in W County were freshwater fish. Forest Production Activities and wood production are contributing to forestry, and their proportions were 75.63% and 24.37%, respectively.

2. Area Demand of Land-Use Types

According to the development planning, the GDP in W County at the end of the 14th Five-Year Plan period is expected to reach RMB 30.92 billion (based on 2015 prices), and the proportions of the three industry types will be 8:39:53. Therefore, the target output value of the primary industry in W County will be RMB 2.47 billion. According to the Statistical Yearbooks from 2016 to 2020, the intermediate input consumption proportion of the primary industry in the county was 52.42%, so the total output value of the primary industry should be RMB 5.19 billion. Substituting this value and the relevant data into Formulas (1)–(5), the area demand for each product contributing to primary industry to realize the GDP was calculated, as shown in Table 6.

It should be pointed out that corn and wheat are rotationally cropped in the county and the planting area of corn is larger than that of wheat, so cultivated land did not carry the output value of wheat. Cattle consume straw in W County and, as straw is an agricultural by-product, no land-use types carried the output value of cattle.

For further clarification, the area demands for cultivated, garden, and forest land to realize the GDP in W County during the 14th Five-Year Plan period were 30,836.32, 25,383.62, and 11,655.09 ha, respectively.

As the target output value of secondary and tertiary industries in W County should be RMB 28.45 billion, according to the development planning, during the 14th Five-Year Plan period, the average annual growth of the output value of unit construction land for these industries should be 7%. Given that the initial value in 2020 was 1,475,805.91 yuan/ha (based on 2015 prices), the target value should be at least 2,069,894.13 yuan/ha. Substituting the above data into Formula (6), we found that the area demands for construction land to realize the GDP was 13,744.66 ha.

#### 4.3.2. Area Demand to Realize Population Development

According to the development planning, the total population of W County in the 14th Five-Year Plan period was expected to reach 580,000, with an urbanization rate of 60%. GM (1,1) models were used to predict the per-capita consumption of primary industry products in the target planning, based on the relevant data in the Statistical Yearbooks from 2016 to 2020. Substituting the above data and the relevant data needed to calculate Formulas (7)–(11), the area demands for cultivated land, garden land, forest land, grassland, and water area to realize population development in the 14th Five-Year Plan period in W County were obtained (Table 7). It should be noted that the quantity of cultivated land products and the grass consumed by animal husbandry products were based on the research results of Hongyu et al. [73].

According to Formula (12), to calculate the construction land area to realize population development, first we need to determine the standards for per-capita urban and rural construction land-use in W County. Based on the official Land Planning data, in the 14th Five-Year Plan period, the standards for urban and rural construction land were 0.017 and 0.015 ha/person, respectively. Therefore, the area of urban and rural construction land required to realize population development was 9396.00 ha. According to the third National Land Survey, the land area for transportation, water conservancy facilities, and other construction was 2200.29 ha. During the 14th Five-Year Plan period, the implementation of key projects increased the area required by 454.30 ha. Therefore, the total area of construction land required to realize population development was 12,050.59 ha.

#### 4.3.3. Area Demand to Realize Socio-Economic Development

Using Formulas (13)–(15) and the previous calculations, we estimated the area demands for cultivated land, garden land, forest land, grassland, water area, and construction land in W County, in order to realize socio-economic development, using LINGO. The parameters were α = 0.05, β = 0.3, and γ = 0.1, and the results are given in Table 8.

### 4.4. Carrying Capacity of Land in W County

Based on the third National Land Survey database, the land-use structure in W County in 2020 is shown in Table 9.

The area of the ecological redline of W County was 41,319.28 ha, of which the area of garden land, forest land, grassland, reservoir surface, and river surface was 3515.54, 24,361.50, 12,733.54, 119.97, and 588.73 ha, respectively. After subtracting this area, the carrying capacities of cultivated land, garden land, forest land, grassland, water area, and construction land in 2020 were 26,276.07, 46,644.96, 19,636.06, 6569.24, 3445.36, and 13,794.72 ha, respectively.

Based on the land planning data, during the 14th Five-Year Plan period, there are several reasons for the changes in land-use types. Transit, water supply, and power supply projects will occupy 96.74, 2.83, and 0.08 ha of cultivated land, respectively, while 2656.98 ha of garden land and 677.32 ha of forest land can be restored to cultivated land. Meanwhile, cultivated land can also be increased by 33.36, 5.08, 90.44, and 35.48 ha through grassland development, bare land development, rural residence integration, and mining land reclamation, respectively. Transit and power supply projects will occupy 65.49 and 5.78 ha of garden land, respectively, while 2656.98 ha of garden land will be restored to cultivated land. Transit, water supply and power supply projects, and fire station construction will occupy 162.58, 0.86, 0.67, and 0.60 ha of forest land, respectively, while 677.32 ha of forest land will be restored to cultivated land. Transit projects will occupy 16.29 ha of grassland and grassland will be increased by 33.36 ha through development. Transit projects will occupy 22.37 ha of river surface. This phenomenon arises when highway land crosses above a river surface; based on the principle that when linear features cross, the upper linear feature maintains continuity, part of the river surface will be reclassified as highway land. Transit projects will occupy 90.83 ha of rural roads, and 55.26 ha of highway land will be renovated without changing the land-use type. The relevant land-use types occupied by transit projects will become highway land, and those occupied by water and power supply projects and fire station construction will become public management and service land. During the planning period, due to the strict control of the ecological redline in W County, no other project will occupy ecological redline land.

After comprehensive calculation and subtracting the ecological redline area, the carrying capacities in 2025 of cultivated land, garden land, forest land, grassland, water area, and construction land of W County in 2025 were 29,675.08, 43,916.71, 18,794.03, 6519.59, 3422.99, and 14,133.92 ha, respectively.

### 4.5. Optimal Land-Use Structure in W County

#### 4.5.1. Equivalence, Regulation, and Comprehensive Factors

The equivalence factors of W County were determined according to equivalent values of ecological services per unit area of Chinese ecosystems determined by Xie et al. [72]. The regulation factors of W County were determined according to the ecological service values per unit area of different land-use types in the province where W County is located, and the research results of Xie et al. After calculation, the equivalence factors of cultivated land, garden land, forest land, grassland, water area, and construction land in W County were 7.90, 19.90, 28.12, 11.67, 45.35, and 1.39, respectively; the regulation factors were 0.64, 0.23, 0.26, 0.15, 0.61, and 0.22, respectively; and the comprehensive factors were 5.06, 4.58, 7.31, 1.75, 27.66, and 0.31, respectively.

#### 4.5.2. Ecological Footprint Generated by Socio-Economic Development

Substituting the demand areas, as well as the equivalence factors and regulation factors, the composition of the ecological footprint generated by socio-economic development in W County was obtained using Formula (16), as detailed in Table 10.

#### 4.5.3. Ecological Carrying Capacity and Its State

Substituting the carrying capacities of land, considering the equivalence factors and regulation factors, the ecological carrying capacity to meet socio-economic development was obtained using Formula (17). Comparing this with the ecological footprint allowed us to estimate the state of the ecological carrying capacity, as shown in Table 11.

It can be seen, from Table 11, that in terms of land-use types, garden land, forest land, grassland, water area, and construction land showed an ecological surplus, while cultivated land showed an ecological deficit.

#### 4.5.4. Optimal Land-Use Structure in W County

Determining the optimal land-use structure should first follow the principle of maintaining ecological balance. It can be seen that the area of cultivated land in W County is in a state of ecological deficit. Therefore, we recommend converting other land-use types to cultivated land. During the conversion, we should maintain land with maximum ecological service values. According to the method mentioned above, we should adjust construction land, grassland, garden land, forest land, and water area successively to supplement cultivated land. Our results showed that converting 1003.12 ha of surplus construction land could supplement the deficit of cultivated land, without having to convert other land-use types. Based on a field investigation, we found that rural residences in W County had room for conversion. By compressing the per-capita standard and clustering its pattern, we could use reduced construction land to supplement cultivated land, thus maintaining ecological balance and maximizing the ecological service values of land in the county. The adjusted ecological carrying capacity to meet socio-economic development and its state in W County are shown in Table 12.

According to the land planning data, during the 14th Five-Year Plan period, the least cultivated areas in W County should reach 20,963.10 ha, and the area of construction land should be controlled at 14,820.33 ha. It can be seen, from Table 12, that the optimal land-use structure can meet the verification targets.

## 5. Discussion

In this paper, we provided methods to optimize county-level land-use structure under the dual background of ecological progress and Multiple Planning Integration; however, there are still limitations and future prospects:

(1) The treatment of the ecological redline area affects the calculation results of the carrying capacity of land, thus affecting the result for land-use structure. In this paper, we suggest that the area of ecological redline be excluded from the carrying capacity of land. The reasons why we adopt this treatment are as follows: on the one hand, the ecological footprint theory suggests that 12% of the actual area in a region should be deducted for biodiversity conservation when calculating the carrying capacity, this is similar to China’s original intention to delimit the ecological redline; on the other hand, many governments in China have stipulated that no one can carry out production or operating activities within the ecological redline, and so does W county. However, in some counties in China, primary land-uses such as cultivated land, garden land, and some construction land types whose dominant function are carrying development are included in the ecological redline. As these situations exist in China, the method in this paper should be further improved when applied to such counties. We consider that, for example, a county might bring cultivated land into the ecological redline, and cultivated land can be used for supporting the socio-economic development of the county; as such, we may analyze this part of cultivated land separately. After clarifying the reasons, we can make relevant decisions. If the reason is for the remediation of pollution in cultivated land, we can prohibit planting or control planting after evaluation; or, if it is just for protection, normal production activities can be carried out, and we can bring that land into the carrying capacity;

(2) In this paper, we propose that the optimal land-use structure should give consideration to the demand area of different land-use types for socio-economic development and the actual carrying capacity; while, at the same time, we should make the land-use structure present the ecological service values as much as possible. Based on these, we propose the rules of conversion between different land-use types. In our case study, there were many adjustable rural residences, which provided an opportunity to supplement the insufficient area of cultivated land. So, this method can be considered feasible in the county. However, if it is difficult to supplement a certain land-use type with deficit status or the total land area of a county is unable to meet the socio-economic development after the conversion of land-use types, non-spatial factors can be used to reduce the dependence of socio-economic development on land. For example, the deficit of cultivated land can be solved through the following method: encourage animal husbandry staff to promote the use of agricultural by-products for feed preparation to support the development of animal husbandry, in order to reduce their dependence on the direct output of cultivated land. This can also prevent the loss of land-use types with higher ecological service values and maintain the total ecological service values provided by land;

(3) The method proposed in this paper has strategic significance for the county to effectively obtain an optimal land-use structure within five years, which belongs to short-term research. Now, many regions have set long-term development targets. However, it is inappropriate to use the proposed method to calculate a detailed land-use structure based on the long-term development targets, because many unforeseen problems may be encountered in the long-term process of socio-economic development. At this time, we should consider the rigidity and elasticity of land-use structure, and our idea is to select some key elements that must be strictly controlled when conducting county-level land use, preliminarily to select the ecological redline, cultivated land, and construction land. We can determine the areas of these key elements based on the relationships between the demand area to meet the long-term target and the supply capacity of the county. The remaining land-use types should then be preliminarily determined every five years, such that the long-term target is broken down in a phased manner, thus dealing with the uncertainty in the process of achieving the long-term target;

(4) This paper constructed models based on the general idea of the ecological footprint theory. Through the calculation of these models, the optimized ideas of taking into consideration the demand from the socio-economic development for land, the carrying capacity of land and the ecological service values of land will be reflected in the results of land-use structure. The application of these models can not only solve the problem that land use cannot be optimized fundamentally, due to the separation of development planning and spatial planning in China, with the global objective contradiction between the demand of human development for land, insufficient land carrying capacity, and land ecological quality degradation; their application can also effectively solve this contradiction and provide technical methods for humanity’s optimal use of land. However, these models are static. Consideration of the development process sometimes has uncertainties, and we can appropriately increase the dynamics of land-use structure. Our idea is that if other regions use these models to calculate the demand area of land for development, they can give elasticity coefficient to a certain socio-economic activity or a certain land-use type in the models, so that some land-use types have a dynamic area.

## 6. Conclusions

At present, China’s land use is faced with the dual background of Multiple Planning Integration and ecological progress, so we attempted to explore methodologies based on the two to optimize land-use structure, focused on the county-level. In accordance with this, an empirical study of W County was carried out, according to the technical route of “set unified targets–calculate demand area–calculate carrying capacity–measure ecological service values–determine the land-use structure”.

(1) The promotion of Multiple Planning Integration on the optimization of land-use structure depends on the integration of development planning and spatial planning. Based on their roles in the formation of the land-use structure, we found that setting unified targets is a critical step. The targets should be set from the two aspects of ecological protection and socio-economic development within five years, which not only responds to the integration of the two planning elements, but also responds to the requirements of ecological progress;

(2) The promotion of ecological progress in the optimization of land-use structure can be reflected in three aspects. First, we should follow ecological priority and give priority to the implementation of the target for ecological protection, that is, the land-use structure should first clarify the ecological redline. Second, the land-use structure should be the result of balancing the demand of land for socio-economic development and the carrying capacity of land. Third, we can bring the ecological service values of the land into the decision-making process pertaining to the land-use structure, through the conversion suggestions of different land-use types, the land-use structure can then present the maximum ecological service values;

(3) Models constructed based on the ecological footprint theory in this paper can quantify the concepts of Multiple Planning Integration and ecological progress. Models used to calculate the demand area of different land-use types can effectively decompose the socio-economic development targets, so as to form a demand structure; models used to calculate the carrying capacity can offer supports for keeping the balance between development demand and the carrying capacity of land; incorporating ecological service values into models in the form of factors can help us make decisions on land-use structure. These models are feasible for application.

## Figures and Tables

**Table 1:** Target system for optimizing county-level land-use structure.

Target	Function
Ecological Redline	Calculating area
Total Population	Calculating area
Urbanization Rate	Calculating area
GDP	Calculating area
Minimum Cultivated Land Area	Verifying area
Total Area of Construction Land	Verifying area

**Table 2:** Land classification standard for optimizing county-level land-use structure.

First-Level Classification	Second-Level Classification	Third-Level Classification	Fourth-Level Classification
Ecological Redline	—	—	—
Socio-economic Development Land	Agricultural Land	Cultivated Land	—
		Garden Land	—
		Forest Land	—
		Grassland	—
		Others	Ditch
			Rural Road
			Pit-pond
			Agricultural Facility Land
			Ridge
	Construction Land	Urban and Rural Construction Land	Residential Land
			Public Management and Service Land
			Commercial Service Land
			Industrial Land
			Urban and Rural Road Land
			Transport Service Station Land
			Salt Pan and Mining Land
			Superfluous Land
		Transportation Land and Water Conservancy Facilities Land	Railway Land
			Rail Transit Land
			Highway Land
			Airport Land
			Port Land
			Pipeline Land
			Water Conservancy Facilities Land
		Other	Special Land
	Water Area and Other Land	Water Area	Reservoir Surface
			River Surface
			Lake Surface
			Glaciers and Permanent Snow
			Inland Beach
			Coastal Beach
			Marshland
		Other Land	

**Table 3:** Formulas for calculating the demand for land to realize GDP.

Formulas	Note	Number
$\begin{array}{ll} S_{cultivated\;land}{\left(GDP\right)}^{\prime } & =\sum_{i=1}^{n}\frac{G_{1}\times r_{a}\times r_{i}}{EP_{i}\times P_{i}}\\ & +\sum_{j=1}^{m}\frac{G_{1}\times r_{d}\times r_{j}}{P_{j}}\times \frac{Q_{ji}}{EP_{i}} \end{array}$	$\mathrm{where}\;S_{cultivated\;land}{\left(GDP\right)}^{\prime }$ represents the area demand for cultivated land (ha); g-th=1, 2, 3,…, nrepresents the i-th$\mathrm{product}\;\mathrm{of}\;\mathrm{cultivated}\;\mathrm{land};\;G_{1}$$\mathrm{represents}\;\mathrm{the}\;\mathrm{target}\;\mathrm{output}\;\mathrm{value}\;\mathrm{of}\;\mathrm{primary}\;\mathrm{industry}\;(\mathrm{yuan});\;r_{a}$$\mathrm{represents}\;\mathrm{the}\;\mathrm{proportion}\;\mathrm{of}\;\mathrm{the}\;\mathrm{agricultural}\;\mathrm{output}\;\mathrm{value}\;(\mathrm{from}\;\mathrm{cultivated}\;\mathrm{land}\;\mathrm{products})\;\mathrm{in}\;\mathrm{the}\;\mathrm{output}\;\mathrm{value}\;\mathrm{of}\;\mathrm{primary}\;\mathrm{industry}\;(\%);\;r_{i}$ represents the proportion of the output value of the i-th$\mathrm{cultivated}\;\mathrm{land}\;\mathrm{product}\;\mathrm{to}\;\mathrm{the}\;\mathrm{agricultural}\;\mathrm{output}\;\mathrm{value}\;(\mathrm{from}\;\mathrm{cultivated}\;\mathrm{land}\;\mathrm{products})\;(\%);\;EP_{i}$ represents the per-unit area yield of the i-th$\;\mathrm{cultivated}\;\mathrm{land}\;\mathrm{product}\;(\mathrm{kg}/\mathrm{ha});\;P_{i}$ represents the price of the i-thcultivated land product (yuan/kg); j= 1, 2, 3,…, m represents the j-th$\;\mathrm{animal}\;\mathrm{husbandry}\;\mathrm{product};\;r_{d}$$\mathrm{represents}\;\mathrm{the}\;\mathrm{proportion}\;\mathrm{of}\;\mathrm{the}\;\mathrm{output}\;\mathrm{value}\;\mathrm{of}\;\mathrm{animal}\;\mathrm{husbandry}\;\mathrm{in}\;\mathrm{the}\;\mathrm{output}\;\mathrm{value}\;\mathrm{of}\;\mathrm{primary}\;\mathrm{industry}\;(\%);\;r_{j}$represents the proportion of the output value of the j-th$\mathrm{animal}\;\mathrm{husbandry}\;\mathrm{product}\;\mathrm{to}\;\mathrm{the}\;\mathrm{output}\;\mathrm{value}\;\mathrm{of}\;\mathrm{animal}\;\mathrm{husbandry}\;(\%);\;P_{j}$represents the price of the j-th$\mathrm{animal}\;\mathrm{husbandry}\;\mathrm{product}\;(\mathrm{yuan}/\mathrm{kg});\;Q_{j}{}_{i}$represents the quantity of the i-thcultivated land product consumed by unit quantity of thej-th animal husbandry product (kg/kg).	(1)
$S_{garden\;land}{\left(GDP\right)}^{\prime }=\sum_{k=1}^{l}\frac{G_{1}\times r_{b}\times r_{k}}{EP_{k}\times P_{k}}$	$\mathrm{where}\;S_{garden\;land}{\left(GDP\right)}^{\prime }$represents the area demand for garden land (ha); k=1, 2, 3,…, lrepresents the k-th$\mathrm{product}\;\mathrm{of}\;\mathrm{garden}\;\mathrm{land};\;r_{b}$$\mathrm{represents}\;\mathrm{the}\;\mathrm{proportion}\;\mathrm{of}\;\mathrm{the}\;\mathrm{agricultural}\;\mathrm{output}\;\mathrm{value}\;(\mathrm{from}\;\mathrm{garden}\;\mathrm{land}\;\mathrm{products})\;\mathrm{in}\;\mathrm{the}\;\mathrm{output}\;\mathrm{value}\;\mathrm{of}\;\mathrm{primary}\;\mathrm{industry}\;(\%);r_{k}$represents the proportion of the output value of the k-th$\mathrm{garden}\;\mathrm{land}\;\mathrm{product}\;\mathrm{to}\;\mathrm{the}\;\mathrm{agricultural}\;\mathrm{output}\;\mathrm{value}\;(\mathrm{from}\;\mathrm{garden}\;\mathrm{land}\;\mathrm{products});\;EP_{k}$$\mathrm{represents}\;\mathrm{the}\;\mathrm{per}\text{-}\mathrm{unit}\;\mathrm{area}\;\mathrm{yield}\;\mathrm{of}\;\mathrm{the}\;k\text{-}\mathrm{th}\;\mathrm{garden}\;\mathrm{land}\;\mathrm{product}\;(\mathrm{kg}/\mathrm{ha});\;P_{k}$ represents the price of the k-th garden land product (yuan/kg).	(2)
$\begin{array}{ll} S_{forest\;land}{\left(GDP\right)}^{\prime } & =\frac{G_{1}\times r_{c}\times r_{h}}{EP_{h}}\\ & +\sum_{t=1}^{s}\frac{G_{1}\times r_{c}\times r_{t}}{EP_{t}\times P_{t}} \end{array}$	$\mathrm{where}\;S_{forest\;land}{\left(GDP\right)}^{\prime }$$\mathrm{represents}\;\mathrm{the}\;\mathrm{area}\;\mathrm{demand}\;\mathrm{for}\;\mathrm{forest}\;\mathrm{land}\;(\mathrm{ha});\;r_{c}$$\mathrm{represents}\;\mathrm{the}\;\mathrm{proportion}\;\mathrm{of}\;\mathrm{the}\;\mathrm{forestry}\;\mathrm{output}\;\mathrm{value}\;\mathrm{in}\;\mathrm{the}\;\mathrm{output}\;\mathrm{value}\;\mathrm{of}\;\mathrm{primary}\;\mathrm{industry}\;(\%);r_{h}$$\mathrm{represents}\;\mathrm{the}\;\mathrm{proportion}\;\mathrm{of}\;\mathrm{the}\;\mathrm{output}\;\mathrm{value}\;\mathrm{of}\;\mathrm{plantation}\;\mathrm{forest}\;\mathrm{production}\;\mathrm{activities}\;\mathrm{in}\;\mathrm{the}\;\mathrm{forestry}\;\mathrm{output}\;\mathrm{value}\;(\%);EP_{h}$represents the unit cost of plantation forest production activities (yuan/ha); t=1, 2, 3,…, srepresents the t-th$\mathrm{product}\;\mathrm{of}\;\mathrm{forest}\;\mathrm{land};\;r_{t}$represents the proportion of the output value of the t-th$\mathrm{forest}\;\mathrm{land}\;\mathrm{product}\;\mathrm{to}\;\mathrm{forestry}\;\mathrm{output}\;\mathrm{value}\;(\%);EP_{t}$represents the per unit area yield of the t-th$\;\mathrm{forest}\;\mathrm{land}\;\mathrm{product}\;(\mathrm{kg}/\mathrm{ha};\;\mathrm{timber},\;m^{3}/\mathrm{ha});\;P_{t}$represents the price of the t-th forest land product (yuan/kg; timber, yuan/m^3^).	(3)
$\begin{array}{ll} S_{grassland}{\left(GDP\right)}^{\prime } & =\sum_{g=1}^{f}(\frac{G_{1}\times r_{d}\times r_{g}}{P_{g}}\\ & \times \frac{Q_{g}}{EP_{\mathrm{grass}}}) \end{array}$	$\mathrm{where}\;S_{grassland}{\left(GDP\right)}^{\prime }$represents the area demand for grassland (ha); g=1, 2, 3,…, frepresents the g-th$\mathrm{product}\;\mathrm{of}\;\mathrm{animal}\;\mathrm{husbandry};\;r_{d}$$\mathrm{represents}\;\mathrm{the}\;\mathrm{proportion}\;\mathrm{of}\;\mathrm{the}\;\mathrm{animal}\;\mathrm{husbandry}\;\mathrm{output}\;\mathrm{value}\;\mathrm{in}\;\mathrm{the}\;\mathrm{output}\;\mathrm{value}\;\mathrm{of}\;\mathrm{primary}\;\mathrm{industry}\;(\%);r_{g}$represents the proportion of the output value of the g-th$\mathrm{animal}\;\mathrm{husbandry}\;\mathrm{product}\;\mathrm{in}\;\mathrm{the}\;\mathrm{animal}\;\mathrm{husbandry}\;\mathrm{output}\;\mathrm{value}\;(\%);P_{g}$represents the price of the g-th$\;\mathrm{animal}\;\mathrm{husbandry}\;\mathrm{product}\;(\mathrm{yuan}/\mathrm{kg});\;Q_{g}$represents the quantity of grass consumed per-unit quantity of the g-th$\;\mathrm{animal}\;\mathrm{husbandry}\;\mathrm{product}\;(\mathrm{kg}/\mathrm{kg});\;EP_{\mathrm{grass}}$ represents the per-unit area yield of the grassland (kg/ha).	(4)
$S_{water\;area}{\left(GDP\right)}^{\prime }=\frac{G_{1}\times r_{e}}{EP_{e}\times P_{e}}$	$\mathrm{where}\;S_{water\;area}{\left(GDP\right)}^{\prime }$$\mathrm{represents}\;\mathrm{the}\;\mathrm{area}\;\mathrm{demand}\;\mathrm{for}\;\mathrm{water}\;\mathrm{area}\;(\mathrm{ha});\;r_{e}$$\mathrm{represents}\;\mathrm{the}\;\mathrm{proportion}\;\mathrm{of}\;\mathrm{the}\;\mathrm{fishery}\;\mathrm{output}\;\mathrm{value}\;\mathrm{in}\;\mathrm{the}\;\mathrm{output}\;\mathrm{value}\;\mathrm{of}\;\mathrm{primary}\;\mathrm{industry}\;(\%);EP_{e}$represents the per-unit area of the catch of aquatic products (kg/ha); Pe represents the price of aquatic products (yuan/kg).	(5)
$S_{construction\;land}{\left(GDP\right)}^{\prime }=\frac{G_{2,3}}{G_{{}_{\mathrm{unit}\;\mathrm{area}}\left(GDP\right)}}$	$\mathrm{where}\;S_{construction\;land}{\left(GDP\right)}^{\prime }$$\;\mathrm{represents}\;\mathrm{the}\;\mathrm{area}\;\mathrm{demand}\;\mathrm{for}\;\mathrm{construction}\;\mathrm{land}\;(\mathrm{ha});\;G_{2,3}$$\;\mathrm{represents}\;\mathrm{the}\;\mathrm{target}\;\mathrm{output}\;\mathrm{value}\;\mathrm{of}\;\sec\mathrm{ondary}\;\mathrm{and}\;\mathrm{tertiary}\;\mathrm{industries}\;(\mathrm{yuan});\;G_{{}_{\mathrm{unit}\;\mathrm{area}}\left(GDP\right)}$ represents the target output value of secondary and tertiary industry per unit area of construction land (yuan/ha).	(6)

**Table 4:** Formulas for calculating the demand for land to realize population development.

Formulas	Note	Number
$\begin{array}{ll} S_{cultivated\;land}{\left(population\right)}^{\prime } & =\frac{P\times u\times c_{1}}{AP_{1}\times F\times D}\\ & +\frac{P\times \left(1-u\right)\times c_{2}}{AP_{1}\times F\times D}\\ & +\sum_{j=1}^{m}\frac{P\times u\times c_{5j}\times c_{5ji}}{AP_{i}}\\ & +\sum_{j=1}^{m}\frac{P\times \left(1-u\right)\times c_{6j}\times c_{6ji}}{AP_{i}} \end{array}$	$\mathrm{where}\;S_{cultivated\;land}{\left(population\right)}^{\prime }$ represents the area demand for cultivated land (ha); Prepresents the target population (person); u$\mathrm{represents}\;\mathrm{the}\;\mathrm{urbanization}\;\mathrm{rate}\;(\%);c_{1}$$\mathrm{represents}\;\mathrm{the}\;\mathrm{grain}\;\mathrm{consumption}\;\mathrm{of}\;\mathrm{urban}\;\mathrm{population}\;(\mathrm{kg}/\mathrm{person}/a);\;AP_{1}$represents the grain yield per unit area (kg/ha/a); Frepresents the multiple cropping index; D$\mathrm{represents}\;\mathrm{the}\;\mathrm{ratio}\;\mathrm{of}\;\mathrm{grain}\;\mathrm{crops}\;\mathrm{to}\;\mathrm{cash}\;\mathrm{crops};\;c_{2}$represents the grain consumption of the rural population (kg/person/a); j=1, 2, 3,…; mrepresents the j-th$\mathrm{animal}\;\mathrm{husbandry}\;\mathrm{product};\;c_{5j}$represents the quantity of the j-th$\mathrm{animal}\;\mathrm{husbandry}\;\mathrm{product}\;\mathrm{consumed}\;\mathrm{by}\;\mathrm{the}\;\mathrm{urban}\;\mathrm{population}\;(\mathrm{kg}/\mathrm{person}/a);\;c_{5ji}$represents the quantity of the i-thcultivated land product consumed by unit quantity of the j-th$\mathrm{animal}\;\mathrm{husbandry}\;\mathrm{product}\;\mathrm{in}\;\mathrm{urban}\;\mathrm{consumption}\;(\mathrm{kg}/\mathrm{kg});\;c_{6j}$represents the quantity of the j-th animal husbandry product consumed by the rural population (kg/ person/a); c6jirepresents the quantity of the i-thcultivated land product consumed by unit quantity of the j-th$\mathrm{animal}\;\mathrm{husbandry}\;\mathrm{product}\;\mathrm{in}\;\mathrm{rural}\;\mathrm{consumption}\;(\mathrm{kg}/\mathrm{kg});\;AP_{i}$represents the per-unit area yield of the i-th cultivated land product (kg/ha/a).	(7)
$\begin{array}{ll} S_{garden\;land}{\left(population\right)}^{\prime } & =\frac{P\times u\times c_{3}}{AP_{2}}\\ & +\frac{P\times \left(1-u\right)\times c_{4}}{AP_{2}} \end{array}$	$\mathrm{where}\;S_{garden\;land}{\left(population\right)}^{\prime }$$\mathrm{represents}\;\mathrm{the}\;\mathrm{area}\;\mathrm{demand}\;\mathrm{for}\;\mathrm{garden}\;\mathrm{land}\;(\mathrm{ha});\;c_{3}$$\mathrm{represents}\;\mathrm{the}\;\mathrm{fruit}\;\mathrm{consumption}\;\mathrm{of}\;\mathrm{the}\;\mathrm{urban}\;\mathrm{population}\;(\mathrm{kg}/\mathrm{person}/a);\;c_{4}$$\mathrm{represents}\;\mathrm{the}\;\mathrm{fruit}\;\mathrm{consumption}\;\mathrm{of}\;\mathrm{the}\;\mathrm{rural}\;\mathrm{population}\;(\mathrm{kg}/\mathrm{person}/a);\;AP_{2}$ represents the per-unit area yield of fruit (kg/ha/a).	(8)
$S_{forest\;land}{\left(population\right)}^{\prime }=\frac{P}{1000}$	$\mathrm{where}\;S_{forest\;land}{\left(population\right)}^{\prime }$ represents the area demand for forest land (ha); 1000 is used to represent the oxygen produced per-unit area of forest land that allows 1000 people to breathe (person/ha).	(9)
$\begin{array}{ll} S_{grassland}{\left(population\right)}^{\prime } & =\sum_{g}^{f}\frac{P\times u\times c_{5g}\times c_{5g_{grass}}}{AP_{{}_{grass}}}\\ & +\sum_{g}^{f}\frac{P\times \left(1-u\right)\times c_{6g}\times c_{6g_{grass}}}{AP_{{}_{grass}}} \end{array}$	$\mathrm{where}\;S_{grassland}{\left(population\right)}^{\prime }$represents the area demand for grassland (ha); g=1, 2, 3,…; frepresents the g-th$\mathrm{animal}\;\mathrm{husbandry}\;\mathrm{product};\;c_{5g}$represents the quantity of the g-th$\;\mathrm{animal}\;\mathrm{husbandry}\;\mathrm{product}\;\mathrm{consumed}\;\mathrm{by}\;\mathrm{the}\;\mathrm{urban}\;\mathrm{population}\;(\mathrm{kg}/\mathrm{person}/a);\;c_{5g_{grass}}$represents the quantity of grass consumed by unit quantity of the g-th$\mathrm{animal}\;\mathrm{husbandry}\;\mathrm{product}\;\mathrm{in}\;\mathrm{urban}\;\mathrm{consumption}\;(\mathrm{kg}/\mathrm{kg});\;AP_{{}_{grass}}$$\mathrm{represents}\;\mathrm{the}\;\mathrm{per}\text{-}\mathrm{unit}\;\mathrm{area}\;\mathrm{yield}\;\mathrm{of}\;\mathrm{grass}\;(\mathrm{kg}/\mathrm{ha}/a);\;c_{6g}$represents the quantity of the g-th$\mathrm{animal}\;\mathrm{husbandry}\;\mathrm{product}\;\mathrm{consumed}\;\mathrm{by}\;\mathrm{the}\;\mathrm{rural}\;\mathrm{population}\;(\mathrm{kg}/\mathrm{person}/a);\;c_{6g_{grass}}$represents the quantity of grass consumed by unit quantity of the g-th animal husbandry product in rural consumption (kg/kg).	(10)
$\begin{array}{ll} S{\;}_{water\;area}{\left(population\right)}^{\prime } & =\frac{P\times u\times c_{7}}{AP_{{}_{water\;area}}}\\ & +\frac{P\times \left(1-u\right)\times c_{8}}{AP_{{}_{water\;area}}} \end{array}$	$\mathrm{where}\;S{\;}_{water\;area}{\left(population\right)}^{\prime }$$\;\mathrm{represents}\;\mathrm{the}\;\mathrm{area}\;\mathrm{demand}\;\mathrm{for}\;\mathrm{water}\;\mathrm{area}\;(\mathrm{ha});\;c_{7}$$\mathrm{represents}\;\mathrm{the}\;\mathrm{quantity}\;\mathrm{of}\;\mathrm{aquatic}\;\mathrm{products}\;\mathrm{consumed}\;\mathrm{by}\;\mathrm{the}\;\mathrm{urban}\;\mathrm{population}\;(\mathrm{kg}/\mathrm{person}/a);\;AP_{{}_{water\;area}}$$\;\mathrm{represents}\;\mathrm{the}\;\mathrm{per}\text{-}\mathrm{unit}\;\mathrm{area}\;\mathrm{for}\;\mathrm{the}\;\mathrm{catching}\;\mathrm{of}\;\mathrm{aquatic}\;\mathrm{products}\;(\mathrm{kg}/\mathrm{ha}/a);\;c_{8}$ represents the quantity of aquatic products consumed by the rural population (kg/person/a).	(11)
$\begin{array}{ll} S_{construction\;land}{\left(population\right)}^{\prime } & =P\times u\times s_{{}_{urban}}\\ & +P\times \left(1-u\right)\times s_{{}_{rural}}\\ & +S_{{}_{others}} \end{array}$	$\mathrm{where}\;S_{construction\;land}{\left(population\right)}^{\prime }$$\;\mathrm{represents}\;\mathrm{the}\;\mathrm{area}\;\mathrm{demand}\;\mathrm{for}\;\mathrm{construction}\;\mathrm{land}\;(\mathrm{ha});\;s_{{}_{urban}}$$\mathrm{represents}\;\mathrm{the}\;\mathrm{standard}\;\mathrm{of}\;\mathrm{urban}\;\mathrm{construction}\;\mathrm{land}\;\mathrm{per}\;\mathrm{capita}\;(\mathrm{ha}/\mathrm{person});\;s_{{}_{rural}}$$\mathrm{represents}\;\mathrm{the}\;\mathrm{standard}\;\mathrm{of}\;\mathrm{rural}\;\mathrm{construction}\;\mathrm{land}\;\mathrm{per}\;\mathrm{capita}\;(\mathrm{ha}/\mathrm{person});\;S_{{}_{others}}$ represents the area of transportation land, water conservancy facilities land, and other construction land (ha).	(12)

**Table 5:** Data for the case study.

Name	Year	Type	Source
Third National Land Survey Database	2020	Vector	Natural Resources Bureau
National Economic and Social Development Planning	2021–2025	Text	Development and Reform Bureau
Statistical Yearbook	2016–2020	Text	Statistical Bureau
Prices of Products in Primary Industry	2015	Text	Price Bureau
Unit Cost of Artificial Forest Production Activities	2015	Text	Forestry Bureau
Land Planning	2021–2035	Text, Vector	Natural Resources Bureau

**Table 6:** Area demand for primary industry products to realize GDP.

Product	Land-Use Type	Area Demand (ha)
Corn (for agricultural development)	Cultivated Land	6164.97
Corn (for pig breeding)	Cultivated Land	5086.94
Corn (for poultry breeding)	Cultivated Land	7234.78
Corn (for egg production)	Cultivated Land	2916.42
Millet	Cultivated Land	1695.19
Sorghum	Cultivated Land	331.17
Soybean	Cultivated Land	806.48
Green Bean	Cultivated Land	69.15
Red Bean	Cultivated Land	57.37
Tuber	Cultivated Land	1136.48
Peanut	Cultivated Land	1426.77
Cotton	Cultivated Land	128.90
Tobacco Leaf	Cultivated Land	279.75
Vegetable	Cultivated Land	3275.55
Watermelon	Cultivated Land	182.76
Cantaloupe	Cultivated Land	19.52
Strawberry	Cultivated Land	24.62
Apple	Garden Land	15,848.14
Pear	Garden Land	249.15
Grape	Garden Land	1743.52
Peach	Garden Land	5731.76
Cherry	Garden Land	1605.33
Walnut	Garden Land	80.69
Chinese Prickly Ash	Garden Land	125.03
Forest Production Activities	Forest Land	11,252.23
Wood	Forest Land	402.86
Sheep	Grassland	3898.75
Fish	Water Area	1574.23

**Table 7:** Area demands for cultivated land, garden land, forest land, grassland, and water to realize population development.

Land-Use Type	Area Demand (ha)
Cultivated Land (cultivated land products)	24,902.85
Cultivated Land (animal husbandry products)	5617.58
Garden Land	1613.73
Forest Land	580.00
Grassland	455.11
Water Area	791.89

**Table 8:** Area demands for realizing socio-economic development.

Land-Use Type	Maximum Area (ha)	Minimum Area (ha)	Area Demand (ha)
Cultivated Land	30,725.65	30,630.83	30,678.20
Garden Land	15,171.23	7093.79	10,374.08
Forest Land	6840.79	3049.26	4567.20
Grassland	2468.18	1322.43	1806.65
Water Area	1282.67	1039.09	1154.47
Construction Land	13,144.31	12,632.38	12,885.80

**Table 9:** Land-use structure in W County in 2020.

Land-Use Type	Area (ha)	Land-Use Type	Area (ha)
Cultivated Land	26,276.07	Railway Land	147.45
Garden Land	50,160.50	Rail Transit Land	0.00
Forest Land	43,997.56	Highway Land	1752.02
Grassland	19,302.78	Airport Land	0.00
Ditches	279.92	Port Land	0.00
Rural Roads	1277.64	Pipeline Land	0.00
Pit-ponds	201.91	Water Conservancy Facilities Land	91.25
Agricultural Facility Land	449.01	Special Land	209.57
Ridge	3853.88	Reservoir Surface	1484.86
Residential Land	7973.32	River Surface	2179.96
Public Management and Service Land	604.87	Lake Surface	0.00
Commercial Service Land	713.28	Glaciers and Permanent Snow	0.00
Industrial Land	1284.56	Inland Beach	7.41
Urban and Rural Road Land	419.95	Coastal Beach	0.00
Transport Service Station Land	49.12	Marshland	0.00
Salt Pan and Mining Land	549.33	Other Land	313.59
Superfluous Land	0.00	Grand Total	163,579.81

**Table 10:** Ecological footprint generated by socio-economic development.

Land-Use Type	Area Demand (ha)	Ecological Footprint (ha)
Cultivated land	30,678.20	155,231.69
Garden land	10,374.08	47,513.29
Forest land	4567.20	33,386.23
Grassland	1806.65	3161.64
Water area	1154.47	31,932.64
Construction land	12,885.80	3994.60
Grand total	61,466.40	275,220.09

**Table 11:** Ecological carrying capacity and its state.

Land-Use Type	Ecological Footprint (ha)	Carrying Capacity (ha)	Ecological Carrying Capacity (ha)	Ecological Surplus/ Ecological Deficit (ha)
Cultivated Land	155,231.69	29,675.08	150,155.90	−5075.79
Garden Land	47,513.29	43,916.71	201,138.53	153,625.24
Forest Land	33,386.23	18,794.03	137,384,.36	103,998.13
Grassland	3161.64	6519.59	11,409.28	8247.64
Water Area	31,932.64	3422.99	94,679.90	62,747.26
Construction Land	3994.60	14,133.92	4381.52	386.92
Grand Total	275,220.09	116,462.32	599,149.50	323,929.41

**Table 12:** Ecological carrying capacity and its state after adjustment.

Land-Use Type	Ecological Footprint (ha)	Carrying Capacity (ha)	Ecological Carrying Capacity (ha)	Ecological Surplus/ Ecological Deficit (ha)
Cultivated Land	155,231.69	30,678.20	155,231.69	0.00
Garden Land	47,513.29	43,916.71	201,138.53	153,625.24
Forest Land	33,386.23	18,794.03	137,384.36	103,998.13
Grassland	3161.64	6519.59	11,409.28	8247.64
Water Area	31,932.64	3422.99	94,679.90	62,747.26
Construction Land	3994.60	13,130.80	4070.55	75.95
Grand Total	275,220.09	116,462.32	603,914.31	328,694.22

## Data Availability

Data sharing not applicable.
